# Prediction model for early neurological deterioration in large artery atherosclerotic stroke

**DOI:** 10.3389/fneur.2026.1868839

**Published:** 2026-07-09

**Authors:** Lele Feng, Chuanzhuo Zhang, Jiayu Zhou, Xinyi Zhang, Jingyi Guo, Benping Zhang

**Affiliations:** 1 Department of Neurology, The Second Affiliated Hospital of Harbin Medical University, Harbin, Heilongjiang, China; 2 Qiqihar Medical University, Qiqihar, Heilongjiang, China

**Keywords:** acute ischemic stroke, early neurological deterioration, large artery atherosclerosis, nomogram, prediction model

## Abstract

**Background:**

To develop and validate a predictive model for early neurological deterioration (END) in acute ischemic stroke due to large artery atherosclerosis.

**Methods:**

We included 433 patients admitted to our hospital between August 2023 and January 2025, randomly split into training (325) and internal validation (108) cohorts (3:1). END was defined as an NIHSS increase of ≥2 points within 7 days. Univariate analysis and LASSO regression selected variables in the training cohort; multivariate logistic regression was used to build the predictive model, which was visualized as a nomogram. Model performance was assessed using ROC curves, calibration curves, and decision curve analysis (DCA).

**Results:**

END occurred in 27.1% of the training cohort and 26.9% of the internal validation cohort. Seven variables were identified: neutrophil count, platelet count, lymphocyte count, fasting plasma glucose, total cholesterol, homocysteine, and D-dimer. Six were independent predictors (*p* < 0.05); fasting plasma glucose showed a trend toward association (*p* = 0.084) and was retained based on LASSO selection. The training cohort AUC was 0.791 (95% CI: 0.730–0.851), with 72.7% sensitivity, 78.5% specificity, and 76.9% accuracy; the internal validation cohort AUC was 0.778 (95% CI: 0.675–0.881), with 75.9% sensitivity, 70.9% specificity, and 72.2% accuracy. Calibration curves showed acceptable fit (mean absolute errors 0.023 and 0.048). Hosmer-Lemeshow tests were all *p* > 0.05. DCA suggested that the model may offer some net benefit across a relatively wide range of threshold probabilities.

**Conclusion:**

The predictive model developed in this study (incorporating neutrophil count, platelet count, lymphocyte count, fasting plasma glucose, total cholesterol, homocysteine, and D-dimer) may serve as a straightforward initial screening tool for early neurological deterioration in patients with acute ischemic stroke due to large artery atherosclerosis. Six of these variables were independent predictors; fasting plasma glucose showed a trend toward association (*p* = 0.084) but was retained in the model based on LASSO selection. The model may facilitate early identification of high-risk patients and provide some reference for individualized treatment decisions.

## 1 Introduction

Acute ischemic stroke is one of the most common life-threatening neurological disorders, with substantial rates of disability and mortality. The primary subtype is large-artery atherosclerosis, with atherosclerotic stenosis or occlusion of the intracranial or extracranial large arteries as its pathological core (1). This patient population frequently experiences early neurological deterioration (END), clinically defined as an increase of ≥2 points on the National Institutes of Health Stroke Scale (NIHSS) within 7 days of onset (2). The pathophysiological mechanisms underlying END are relatively complex and may involve infarct expansion, collateral circulation failure, and reperfusion injury; once it occurs, END is often associated with unfavorable outcomes (3, 4). Therefore, early identification of high-risk populations may be clinically significant for improving overall prognosis.

Previous studies have explored variables associated with early neurological deterioration in patients with acute ischemic stroke; however, most have not performed stratified analyses based on stroke etiological subtypes, which may limit the specificity of their findings. Furthermore, few END prediction models systematically integrate laboratory indicators with clinical parameters, and no specific prediction tools are currently available for patients with large artery atherosclerotic acute ischemic stroke (5). This study aimed to develop and internally validate a clinical prediction model for END in this specific population, with the expectation of providing some reference for the formulation of early screening and intervention strategies in clinical practice.

## 2 Materials and methods

### 2.1 Study population

Patients with acute ischemic stroke who were admitted to the Second Affiliated Hospital of Harbin Medical University’s Department of Neurology between August 2023 and January 2025 made up the study population. Inclusion criteria: Age ≥ 18 years; diagnosis of large-artery atherosclerosis stroke based on the TOAST classification (6); diagnosis of acute ischemic stroke verified by diffusion-weighted imaging (DWI) of the head (7). Exclusion criteria: Patients with severe functional impairment of essential organs, such as the kidneys, liver, or heart; patients with concomitant malignant malignancies.

A training cohort (325 patients) and an internal validation cohort (108 patients) were randomly selected from a total of 433 eligible patients in a 3:1 ratio. The Ethics Committee of our hospital examined and approved this study (Ethics Approval No.: YJSKY2025-497), and all study procedures comply with medical ethical standards.

### 2.2 Data collection

The hospital’s electronic medical record system was used to retrospectively gather all of the enrolled patients’ clinical data.

General Information: This comprises the patient’s age, gender, and medical history of underlying conditions, including diabetes and hypertension.

Laboratory Tests: Within 24 h after the patient’s admission, blood samples must be taken on an empty stomach for testing. C-reactive protein (CRP), fasting plasma glucose, total cholesterol, low-density lipoprotein, lipoprotein (a), homocysteine, alkaline phosphatase, albumin, total serum bilirubin, plasma fibrinogen, D-dimer, white blood cell count, neutrophil count, lymphocyte count, monocyte count, platelet count, and mean platelet volume.

### 2.3 Definitions and outcome measures

Early neurological deterioration (END) is defined as a patient’s National Institutes of Health Stroke Scale (NIHSS) total score rising by at least two points from the baseline score at admission within 7 days of commencement. Two neurologists independently evaluated each NIHSS score; if there was disagreement, the final score was decided by mutual consultation.

### 2.4 Statistical analysis

**Figure 1:**
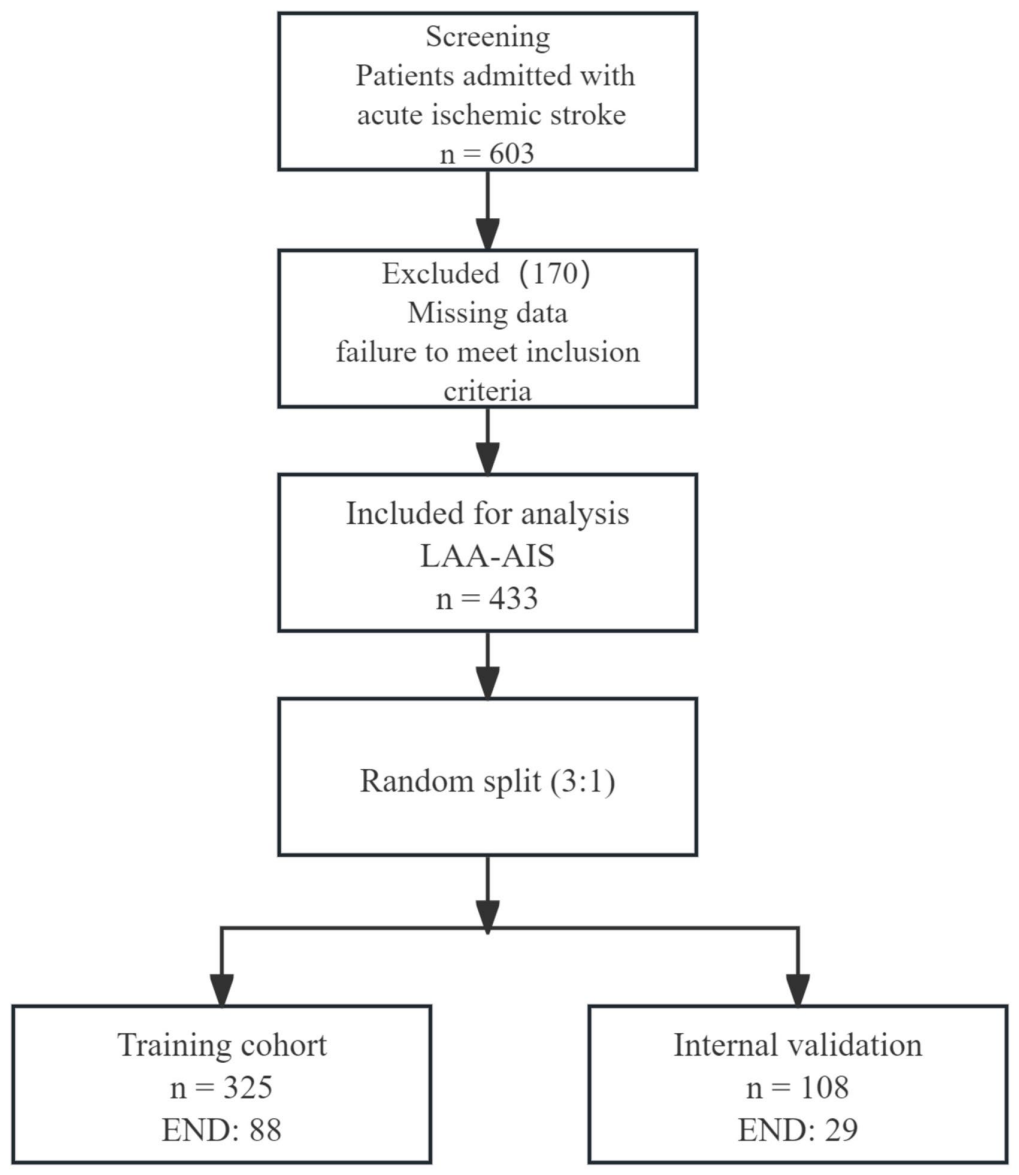
Flowchart of patient selection.

Patient flow is depicted in Figure 1 (screened *n* = 603; eliminated *n* = 170 due to missing data, failure to meet inclusion criteria, etc.; included *n* = 433). Imputation was not required because none of the 21 potential variables had missing data. We used SPSS 25.0 and R 4.1.0 for data analysis. The dataset was divided 3:1 into training (*n* = 325) and internal validation (*n* = 108) cohorts. Normally distributed continuous variables are reported as mean ± SD and compared using independent t-tests; non-normal variables as median (IQR) and compared using Wilcoxon rank-sum tests. Categorical variables are reported as counts (%) and compared using chi-square or Fisher’s exact tests as appropriate. For model development, variable selection drew on clinical relevance, routine lab availability, and LASSO regression with 10-fold cross-validation. First, we used ROC analysis to dichotomize each laboratory indicator, and then we used univariate logistic regression to look for correlations with END. Seven variables remained after the LASSO regression model was fed variables with *p* < 0.05 (8). The nomogram was then created by including these seven variables into a multivariate logistic regression model (9). In the training and internal validation cohorts, we used ROC curves, DCA, and calibration curves to evaluate the model’s prediction performance (10). Calibration curves were produced by bootstrap resampling (B = 1,000) on the training cohort and adjusted for optimism (apparent AUC 0.791, optimism 0.017, corrected AUC 0.774). This indicated negligible overfitting and was consistent with the split-sample finding (0.778). Finally, we performed two sensitivity analyses: (1) all 21 variables entered LASSO directly (lambda. 1se) without univariate screening, compared with the main model; (2) a continuous-variable alternative, with neutrophil count, D-dimer, and lymphocyte count (nonlinearity *p* = 0.007) fit via restricted cubic splines (4 knots) and others left linear.

## 3 Result

### 3.1 Comparing the training and internal validation cohorts’ baseline data

This study included 433 patients with large artery atherosclerotic acute ischemic stroke. They were randomly divided into a training cohort (*n* = 325) and an internal validation cohort (*n* = 108) at a ratio of 3:1. No statistically significant differences were detected between the two groups in terms of sex, age, prevalence of hypertension, or prevalence of diabetes (all *p* > 0.05), as shown in Table 1.

**Table 1:** Baseline characteristics of training and validation cohorts.

Variable	Training cohort (*N* = 325)	Validation cohort (*N* = 108)	χ^2^/z/t	*p*-value
Sex			0.50	0.4786
Male	205 (63.08)	64 (59.26)		
Female	120 (36.92)	44 (40.74)		
Age, years	64 (56, 70)	66(56.25, 71)	−0.87	0.3837
Hypertension			0.49	0.4852
No	135 (41.54)	49 (45.37)		
Yes	190 (58.46)	59 (54.63)		
Diabetes			0.20	0.6513
No	215 (66.15)	74 (68.52)		
Yes	110 (33.85)	34 (31.48)		

Continuous variables, median (IQR); categorical variables, n (%).

### 3.2 Comparing the training cohort’s case group and control group

Baseline characteristics were compared between the case group (*n* = 88) and the control group (*n* = 237) within the training cohort. The results showed that no statistically significant differences were detected between the two groups in terms of sex, age, or prevalence of diabetes (all *p* > 0.05). Although there was an increase in the case group’s prevalence of hypertension, the difference was not statistically significant (*p* = 0.0557). For metabolic biomarkers, patients in the case group had higher fasting plasma glucose, total cholesterol, homocysteine, and low-density lipoprotein cholesterol relative to controls (all *p* < 0.05). When examining coagulation and inflammatory parameters, the case group showed elevated D-dimer, white blood cell, and neutrophil counts, alongside lower lymphocyte and platelet counts (all *p* < 0.001). Liver function and nutritional markers—including albumin, alkaline phosphatase, and total bilirubin—did not differ meaningfully between the two groups, as shown in Supplementary Table 1 (all *p* > 0.05).

### 3.3 Analysis of univariate logistic regression

Univariate logistic regression was performed for preliminary screening of variables associated with early neurological deterioration (END). The findings were as follows: fasting plasma glucose≥ 5.69 (OR = 1.68, 95% CI: 1.03–2.76, *p* = 0.0388), total cholesterol ≥ 4.06 (OR = 2.20, 95% CI: 1.25–3.85, *p* = 0.0061), LDL-cholesterol ≥ 2.8 (OR = 1.74, 95% CI: 1.05–2.87, *p* = 0.0319), homocysteine ≥ 15.7 (OR = 2.00, 95% CI: 1.14–3.54, *p* = 0.0165), D-dimer ≥ 146.22 (OR = 3.33, 95% CI: 1.99–5.57, *p* < 0.0001), white blood cell count ≥ 10.55 (OR = 3.81, 95% CI: 1.82–7.94, *p* = 0.0004), and neutrophil count ≥ 9.05 (OR = 44.65, 95% CI: 5.77–345.26, *p* = 0.0003) were associated with the outcome event; A lymphocyte count ≥ 1.27 (OR = 0.27, 95% CI: 0.15–0.49, *p* < 0.0001) and a platelet count ≥ 207.5 (OR = 0.33, 95% CI: 0.19–0.55, *p* < 0.0001) were inversely associated with the outcome event. The remaining variables showed no statistically significant association with the outcome events (all *p* > 0.05), as shown in Supplementary Table 2.

### 3.4 LASSO regression analysis

**Figure 2:**
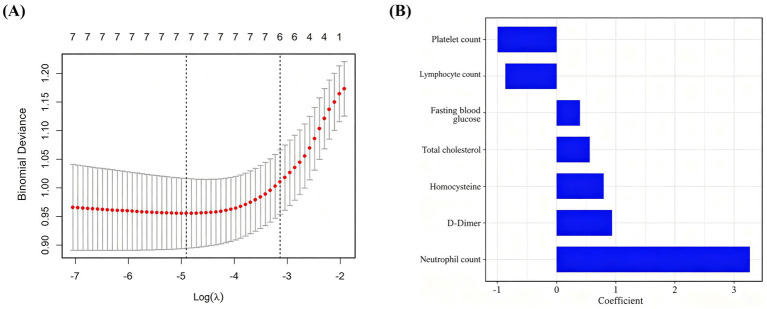
LASSO regression analysis. **(A)** Cross-validation curve; **(B)** selected variables and coefficients at optimal lambda.

*p* < 0.05 variables from the univariate analysis were included in the LASSO regression model for additional screening. Neutrophil count, platelet count, lymphocyte count, fasting plasma glucose, total cholesterol, homocysteine, and D-dimer are the seven variables that were ultimately found using 10-fold cross-validation (Figures 2A,B). The optimal penalty parameter *λ* was found to be 0.007424435 (11).

### 3.5 Analysis of multivariate logistic regression

**Figure 3:**
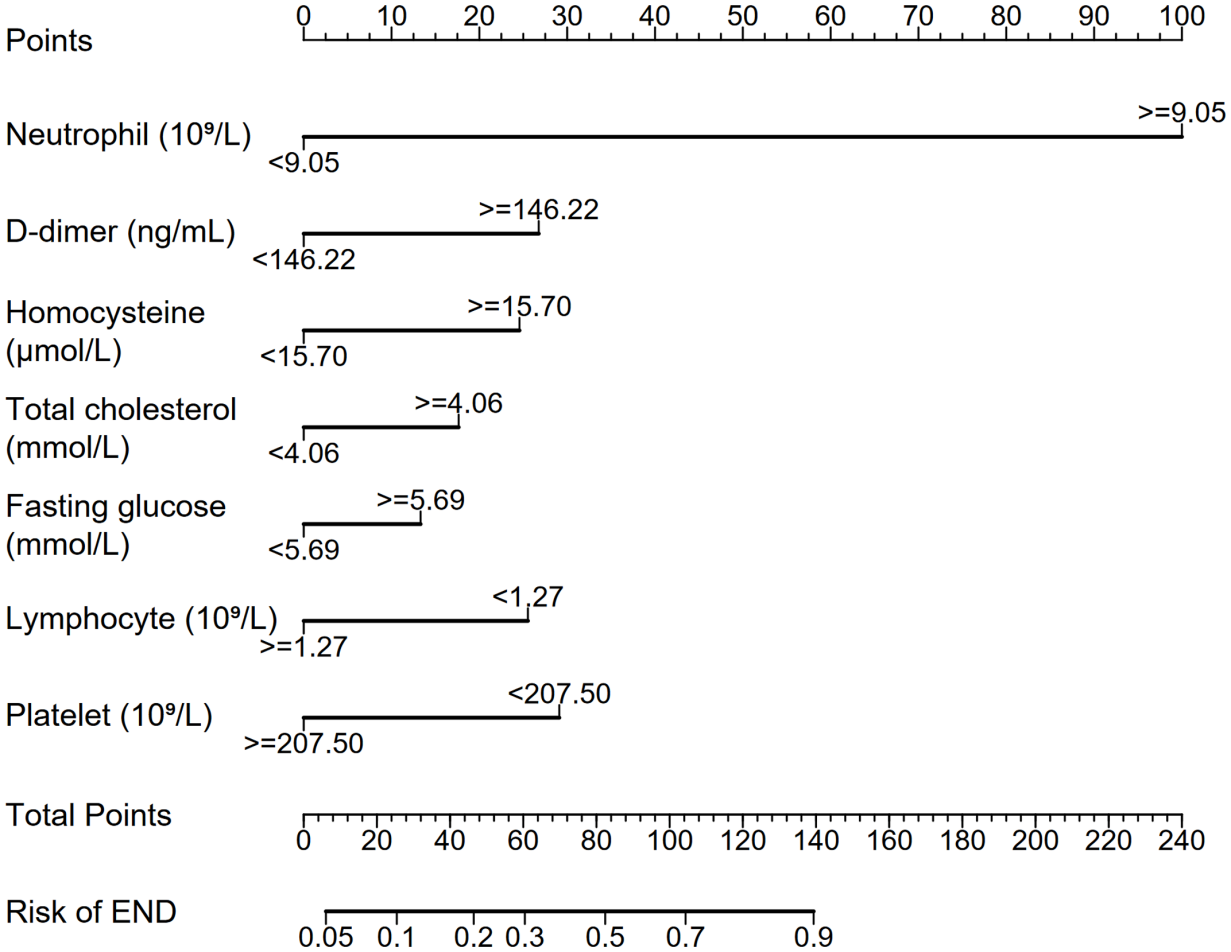
Nomogram for early neurological deterioration prediction.

A multivariate logistic regression model using the seven variables found by LASSO regression revealed that six of these indicators were independently associated with the occurrence of END (*p* < 0.05); fasting plasma glucose showed a trend toward association (*p* = 0.084) and was retained based on LASSO selection. Table 2 displays the odds ratios (ORs) and regression coefficients for each variable. The findings of multivariate regression were used to create a nomogram, where each variable was given a score. Higher scores suggest a potentially higher risk of END, and the total score can be used to estimate the probability of END occurrence (Figure 3).

**Table 2:** Multivariate logistic regression analysis in the training cohort.

Variable	β	SE	Wald χ^2^	*p*-value	OR (95% CI)
Neutrophil count(×10^9/L) ≥ 9.05	3.86	1.12	11.9	0.0006	47.34 (5.29, 423.58)
D-dimer (ng/mL)≥146.22	1.03	0.3	12.05	0.0005	2.81 (1.57, 5.03)
Homocysteine (μ mol/L), ≥ 15.7	0.95	0.35	7.51	0.0061	2.58 (1.31, 5.08)
Total cholesterol (mmol/L), ≥ 4.06	0.68	0.33	4.34	0.0373	1.98 (1.04, 3.75)
Fasting plasma glucose (mmol/L), ≥ 5.69	0.51	0.3	2.99	0.0840	1.67 (0.93, 2.99)
Lymphocyte count (×10^9^/L), ≥ 1.27	−0.98	0.36	7.62	0.0058	0.37 (0.19, 0.75)
Platelet count (×10^9^/L), ≥ 207.5	−1.12	0.32	12.7	0.0004	0.33 (0.18, 0.60)

Ref, reference; β, regression coefficient; SE, standard error; OR, odds ratio; CI, confidence interval.

### 3.6 Regression equation and risk calculation

The final multivariable logistic regression model incorporated seven variables. The logistic regression equation is as follows:

$\begin{array}{l} \text{Logit}(P)=-1.071+3.858\times \text{neutrophil count}+1.032\times D\\ -\text{dimer}+0.948\times \text{homocysteine}+0.681\times \text{total cholesterol}\\ +0.513\times \text{fasting plasma glucose}-0.985\times \text{lymphocyte count}\\ -1.123\times \text{platelet count} \end{array}$ .

In the presented formula, P indicates the predicted probability of early neurological deterioration (END), with an intercept (*β*₀) of −1.071. All predictive variables were dichotomized using optimal cut-off points, whereby scores were defined as 1 for values at or above the threshold and 0 for values below it. The optimal cut-off values were 9.05 × 10^9^/L for neutrophil count, 146.22 ng/mL for D-dimer, 15.70 μmol/L for homocysteine, 4.06 mmol/L for total cholesterol, 5.69 mmol/L for fasting plasma glucose, 1.27 × 10^9^/L for lymphocyte count, and 207.50 × 10^9^/L for platelet count. Individual END risk was estimated using a standard logistic transformation.


$$P=\frac{1}{1+e^{-\text{logit}}}$$

We demonstrate model performance with a clinical example of a 65-year-old patient whose lab findings included neutrophil count 11.2 × 10^9^/L, D-dimer 180 ng/mL, homocysteine 12 μmol/L, total cholesterol 4.5 mmol/L, fasting plasma glucose 6.2 mmol/L, lymphocyte count 1.0 × 10^9^/L,and platelet count 190 × 10^9^/L. Calculation using our regression coefficients returned a logit score of 5.013 (−1.071 + 3.858 + 1.032 + 0 + 0.681 + 0.513). Plugging this score into the prediction equation yields $P=\frac{1}{1+e^{-5.013}}$ ​ ≈ 0.9934 (99.3%). For routine clinical use, providers may also estimate END risk visually via the nomogram in Figure 3.

### 3.7 Nomogram model internal validation and assessment

**Figure 4:**
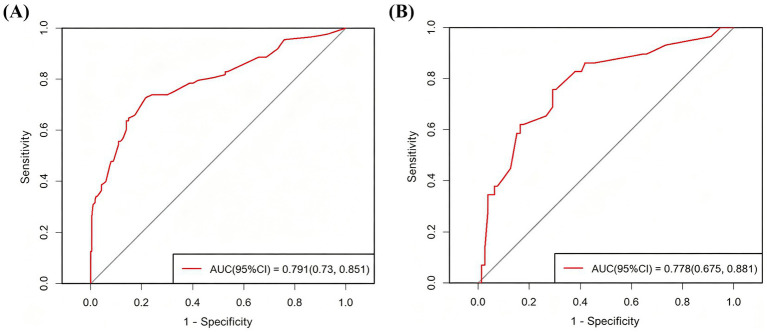
ROC curves for the training cohort **(A)** and validation cohort **(B)**. **(A)** Training cohort (AUC = 0.791, 95% CI: 0.730–0.851); **(B)** validation cohort (AUC = 0.778, 95% CI: 0.675–0.881).

**Figure 5:**
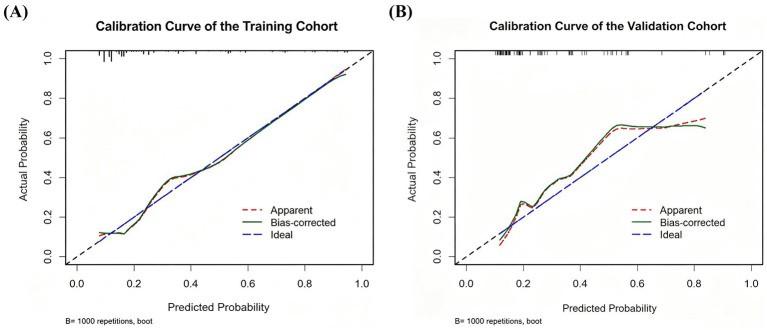
Calibration curves for the training cohort **(A)** and validation cohort **(B)**. **(A)** Training cohort (MAE = 0.023); **(B)** validation cohort (MAE = 0.048).

**Figure 6:**
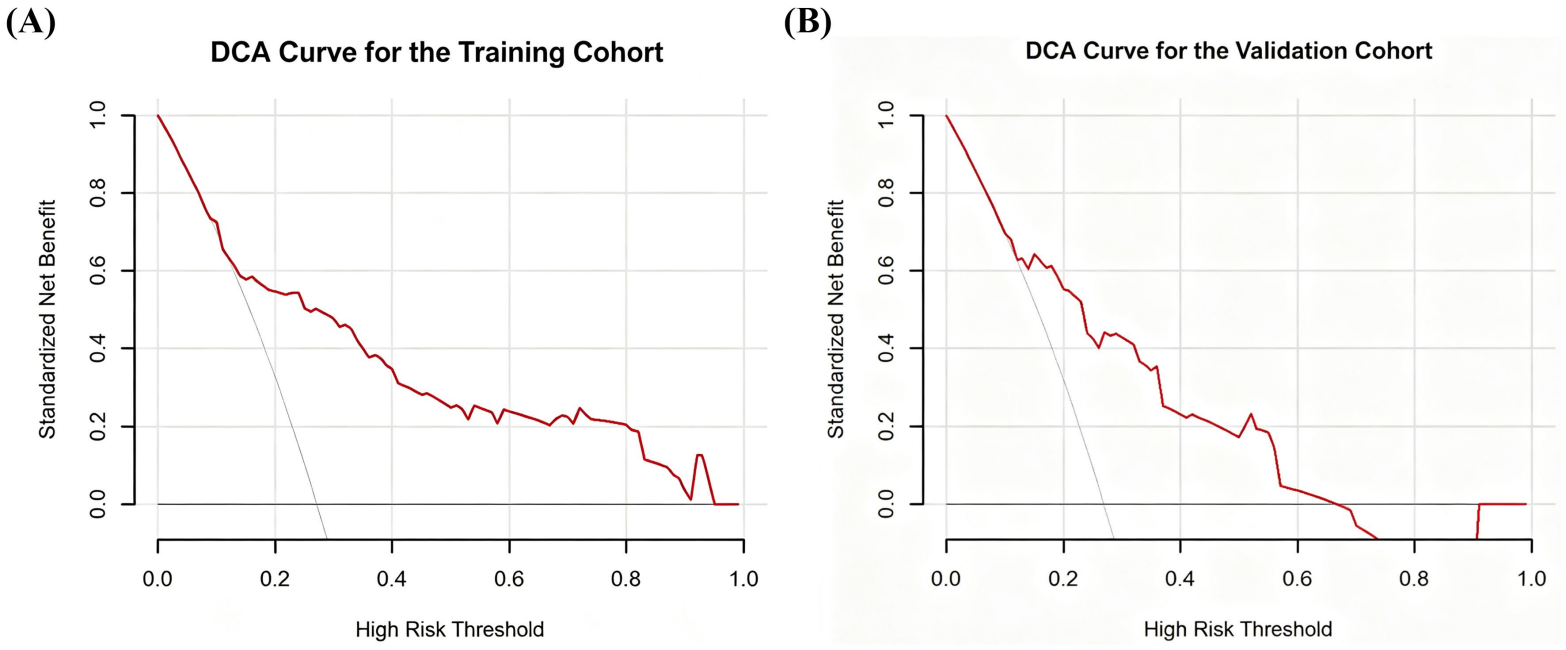
DCA curves for the training cohort **(A)** and validation cohort **(B)**. **(A)** Training cohort; **(B)** validation cohort.

The training cohort’s AUC was 0.791 (95% CI, 0.730–0.851), whereas the internal validation cohort’s was 0.778 (95% CI, 0.675–0.881; Figures 4A,B). The comparable AUC values and overlapping confidence intervals suggest reasonable discriminative ability for identifying patients at risk of END after large-artery atherosclerotic stroke; Bootstrap-based calibration analysis indicates that there is a certain degree of consistency between the predicted probabilities and the observed outcomes in both cohorts (Figures 5A,B). The training cohort had a calibration slope of 1.000 and an intercept of 0.000. The validation cohort showed a slope of 0.835 and an intercept of −0.159, with mean absolute errors of 0.023 (*n* = 325) and 0.048 (*n* = 108), respectively; Decision curve analysis (Figures 6A,B) indicated that the established prediction model appeared to confer favorable net clinical benefit across a broad range of threshold probabilities, providing preliminary support for its potential clinical utility (12). We carried out two sensitivity analyses. In the first, we refitted LASSO regression across all 21 candidate predictors, which retained D-dimer, neutrophil count, lymphocyte count, and platelet count—largely consistent with the predictors used in our primary model. With a validated AUC of 0.758, the resulting alternative model did not statistically vary from the primary model (AUC = 0.778; DeLong’s test, *p* = 0.71). Using untransformed continuous covariates, we constructed a different model for our second sensitivity assessment. Its validated AUC was 0.727, and there was no statistically significant change from the original model (AUC = 0.778; DeLong’s test, *p* = 0.23). In summary, our nomogram demonstrated acceptable discrimination and calibration in the internal validation cohort. Although the model showed reasonable internal consistency, its generalizability to routine clinical practice remains uncertain and requires further external validation.

## 4 Discussion

We restricted our analysis to patients suffering from acute ischemic stroke secondary to large artery atherosclerosis (LAA). Our team built and validated an END prediction model to aid clinicians in pinpointing high-risk cases early and designing personalized treatment plans. LAA-driven acute stroke commonly features bulky intracranial thrombus alongside major large-vessel stenosis or occlusion. Given marked interindividual variation in collateral circulation reserve, patients with this stroke subtype carry a notably higher likelihood of developing END compared with other causal subtypes of AIS (13, 14). In addition to considerably raising patient mortality and disability rates (15), early neurological deterioration also lengthens hospital stays and raises the cost of healthcare (16). Predicting which LAA-AIS patients will develop END early remains a key unmet clinical need. We leveraged LASSO regression to screen seven readily available clinical indices and build an END prediction nomogram. Internal validation findings suggest acceptable prognostic performance, indicating that this model may have potential value for rapid bedside identification of END-prone patients with LAA-AIS.

Neutrophil elevation has been associated with increased END susceptibility in LAA-AIS. Activated neutrophils may contribute to neuroinflammation, blood–brain barrier disruption, and thrombotic progression (17). Lower lymphocyte levels commonly reflect the development of post-stroke immunosuppression among patients with LAA-AIS (18), and together these two factors indicate that inflammation-immune imbalance may be involved in the development of END (19). Increased D-dimer levels have been associated with thrombotic activity and may reflect progressive thrombus growth as well as altered coagulation and fibrinolysis in LAA stroke (20). Homocysteine and total cholesterol have been implicated as metabolic risk factors that may contribute to vascular endothelial injury and plaque instability, processes potentially associated with an increased likelihood of END following LAA-AIS (21, 22). The ischemic penumbra may be more severely damaged by elevated fasting plasma glucose (23). Low platelet counts are more likely to indicate a hyperactive thrombus state and are linked to a higher risk of END, which is believed to be caused by thrombus consumption, post-stroke immunosuppression, or previous antiplatelet medication (24, 25).

The predictive model demonstrated acceptable discriminatory performance (AUC: 0.791 in training; 0.778 in internal validation). Decision curve analysis indicated potential clinical utility, with potential net benefit across a range of threshold probabilities (26). Calibration analyses produced MAEs of 0.023 and 0.048, suggesting acceptable alignment between predicted risks and observed outcomes. Unlike earlier END prediction models developed for unselected ischemic stroke cohorts, we restricted study inclusion to LAA patients to reduce bias stemming from heterogeneous stroke etiologies (27). Meanwhile, the use of LASSO regression for variable selection may improve predictive accuracy and stability, and may also reduce the risk of model overfitting (28). The nomogram is clear and concise, facilitating clinicians’ understanding and reference.

This study has several limitations. First, the single-center design with internal validation only limits generalizability, as the validation subset was derived from random partitioning of the same cohort rather than an independent external dataset. Multicenter external validation is warranted to confirm our findings (29). Second, the training set included 88 events, yielding an EPV of 12.6 for the final 7-variable model. However, if calculated based on the 21 candidate variables screened during model development, the EPV would be 4.2, raising concern for potential overfitting given the relatively modest sample size. Third, baseline characterization was limited to variables with high data completeness; those with substantial missingness were excluded. Furthermore, the final model relied solely on admission laboratory parameters. Several prognostically relevant clinical variables—including baseline NIHSS score, infarct location and volume, arterial stenosis severity, collateral circulation status, acute reperfusion therapy, onset-to-admission time, and prior antiplatelet or anticoagulant use—could not be incorporated due to incomplete retrospective data. These omissions may limit the model’s comprehensiveness and external generalizability (30). Fourth, previous studies reported END rates of 15–20%, whereas our cohort showed a higher incidence (27.1%). This discrepancy likely reflects three methodological differences: our exclusive focus on large artery atherosclerotic stroke, a sensitive threshold (≥2-point NIHSS increase), and enrolment limited to the first 7 days post-stroke. Consequently, cross-population comparisons should be interpreted cautiously (31). Finally, while dichotomizing continuous variables is debated, it facilitates clinical risk stratification. The neutrophil cutoff was ≥9.05 × 10^9^/L; few patients exceeded this threshold, yielding a wide OR estimate (47.34, 95% CI 5.29–423.58). Sensitivity analyses confirmed stable continuous-model performance (bootstrap optimism index, 0.017) with comparable AUCs (*p* = 0.23), suggesting this OR reflects sparse data near the cutoff rather than overfitting. It should not be generalized for routine risk estimation.

In summary, we developed a predictive model for early neurological deterioration (END) after large artery atherosclerotic stroke using routine laboratory biomarkers. Internal validation suggested acceptable discriminative performance, which may support admission risk stratification. However, the single-center retrospective design and lack of external validation warrant cautious interpretation. Further multicenter prospective studies are needed to assess external validity and clinical utility. Future work may include validation across diverse centers and exploration of real-time monitoring and neuroimaging biomarkers for model refinement.

## Data Availability

The datasets presented in this article are not readily available because due to patient privacy protection and institutional data policies, only anonymized data can be provided upon reasonable request. Requests to access the datasets should be directed to Benping Zhang, Zhangbp06@126.com.
