# Supplementary material for: Prediction model for early neurological deterioration in large artery atherosclerotic stroke
Source: Front Neurol. 2026 Jul 9;17:1868839. doi: 10.3389/fneur.2026.1868839 (PMC13391389; doi:10.3389/fneur.2026.1868839)
Supplement: Supplementary file 1 [file Table_1.docx]

Supplementary Material

## Supplementary Tables

**Supplementary Table1.** **Comparison of baseline characteristics between case and control groups in the training cohort. Categorical variables, n (%).**

| Variable | Control Group(N=237) | Case Group (N=88) | χ² | P-value |
| --- | --- | --- | --- | --- |
| Sex |  |  | 2.83 | 0.0923 |
| Male | 156 (65.82) | 49 (55.68) |  |  |
| Female | 81 (34.18) | 39 (44.32) |  |  |
| Age (years) |  |  | 0.66 | 0.4148 |
| <60.5 | 98 (41.35) | 32 (36.36) |  |  |
| ≥60.5 | 139 (58.65) | 56 (63.64) |  |  |
| Hypertension |  |  | 3.66 | 0.0557 |
| No | 106 (44.73) | 29 (32.95) |  |  |
| Yes | 131 (55.27) | 59 (67.05) |  |  |
| Diabetes |  |  | 1.59 | 0.2069 |
| No | 152 (64.14) | 63 (71.59) |  |  |
| Yes | 85 (35.86) | 25 (28.41) |  |  |
| C-reactive protein (mg/L) |  |  | 2.7 | 0.1003 |
| <3.68 | 158 (66.67) | 50 (56.82) |  |  |
| ≥3.68 | 79 (33.33) | 38 (43.18) |  |  |
| Fasting plasma glucose (mmol/L) |  |  | 4.31 | 0.0379 |
| <5.69 | 133 (56.12) | 38 (43.18) |  |  |
| ≥5.69 | 104 (43.88) | 50 (56.82) |  |  |
| Total cholesterol (mmol/L) |  |  | 7.72 | 0.0055 |
| <4.06 | 93 (39.24) | 20 (22.73) |  |  |
| ≥4.06 | 144 (60.76) | 68 (77.27) |  |  |
| Low-density lipoprotein cholesterol (mmol/L) |  |  | 4.65 | 0.031 |
| <2.8 | 118 (49.79) | 32 (36.36) |  |  |
| ≥2.8 | 119 (50.21) | 56 (63.64) |  |  |
| Lipoprotein(a) (mg/L) |  |  | 1.83 | 0.1763 |
| <42.95 | 154 (64.98) | 50 (56.82) |  |  |
| ≥42.95 | 83 (35.02) | 38 (43.18) |  |  |
| Homocysteine (μmol/L) |  |  | 5.88 | 0.0153 |
| <15.7 | 196 (82.7) | 62 (70.45) |  |  |
| ≥15.7 | 41 (17.3) | 26 (29.55) |  |  |
| Alkaline phosphatase (U/L) |  |  | 1.33 | 0.2481 |
| <104.5 | 165 (69.62) | 67 (76.14) |  |  |
| ≥104.5 | 72 (30.38) | 21 (23.86) |  |  |
| Albumin (g/L) |  |  | 0.36 | 0.548 |
| <43.25 | 115 (48.52) | 46 (52.27) |  |  |
| ≥43.25 | 122 (51.48) | 42 (47.73) |  |  |
| Total serum bilirubin (μmol/L) |  |  | 2.69 | 0.1011 |
| <13.95 | 163 (68.78) | 52 (59.09) |  |  |
| ≥13.95 | 74 (31.22) | 36 (40.91) |  |  |
| Plasma fibrinogen (g/L) |  |  | 0.51 | 0.4746 |
| <3.72 | 194 (81.86) | 75 (85.23) |  |  |
| ≥3.72 | 43 (18.14) | 13 (14.77) |  |  |
| D-dimer (ng/mL) |  |  | 22.14 | <0.0001 |
| <146.22 | 150 (63.29) | 30 (34.09) |  |  |
| ≥146.22 | 87 (36.71) | 58 (65.91) |  |  |
| White blood cell count (×10⁹/L) |  |  | 14.04 | 0.0002 |
| <10.55 | 222 (93.67) | 70 (79.55) |  |  |
| ≥10.55 | 15 (6.33) | 18 (20.45) |  |  |
| Neutrophil count (×10⁹/L) |  |  | 31.53 | <0.0001 |
| <9.05 | 236 (99.58) | 74 (84.09) |  |  |
| ≥9.05 | 1 (0.42) | 14 (15.91) |  |  |
| Lymphocyte count (×10⁹/L) |  |  | 20.63 | <0.0001 |
| <1.27 | 29 (12.24) | 30 (34.09) |  |  |
| ≥1.27 | 208 (87.76) | 58 (65.91) |  |  |
| Monocyte count (×10⁹/L) |  |  | 3.03 | 0.0816 |
| <0.36 | 128 (54.01) | 57 (64.77) |  |  |
| ≥0.36 | 109 (45.99) | 31 (35.23) |  |  |
| Platelet count (×10⁹/L) |  |  | 18.12 | <0.0001 |
| <207.5 | 47 (19.83) | 38 (43.18) |  |  |
| ≥207.5 | 190 (80.17) | 50 (56.82) |  |  |
| Mean platelet volume (f L) |  |  | 2.79 | 0.0949 |
| <10.05 | 91 (38.4) | 25 (28.41) |  |  |
| ≥10.05 | 146 (61.6) | 63 (71.59) |  |  |

**Supplementary Table2. Univariate logistic regression analysis of selected variables in the training cohort. Ref, reference; β, regression coefficient; SE, standard error; OR, odds ratio; CI, confidence interval.**

|  | β | SE | Wald χ² | P-value | OR (95% CI) |
| --- | --- | --- | --- | --- | --- |
| Sex |  |  |  |  |  |
| Male | Ref |  |  |  |  |
| Female | 0.43 | 0.25 | 2.82 | 0.0934 | 1.53 (0.93,2.52) |
| Age, years |  |  |  |  |  |
| <60.5 | Ref |  |  |  |  |
| ≥60.5 | 0.21 | 0.26 | 0.66 | 0.4152 | 1.23 (0.74,2.05) |
| Hypertension |  |  |  |  |  |
| No | Ref |  |  |  |  |
| Yes | 0.5 | 0.26 | 3.63 | 0.0568 | 1.65 (0.99,2.75) |
| Diabetes |  |  |  |  |  |
| No | Ref |  |  |  |  |
| Yes | -0.34 | 0.27 | 1.59 | 0.208 | 0.71 (0.42,1.21) |
| C-reactive protein (mg/L) |  |  |  |  |  |
| <3.68 | Ref |  |  |  |  |
| ≥3.68 | 0.42 | 0.26 | 2.68 | 0.1013 | 1.52 (0.92,2.51) |
| Fasting plasma glucose (mmol/L) |  |  |  |  |  |
| <5.69 | Ref |  |  |  |  |
| ≥5.69 | 0.52 | 0.25 | 4.27 | 0.0388 | 1.68 (1.03,2.76) |
| Total cholesterol (mmol/L) |  |  |  |  |  |
| <4.06 | Ref |  |  |  |  |
| ≥4.06 | 0.79 | 0.29 | 7.51 | 0.0061 | 2.2 (1.25,3.85) |
| Low-density lipoprotein cholesterol (mmol/L) |  |  |  |  |  |
| <2.8 | Ref |  |  |  |  |
| ≥2.8 | 0.55 | 0.26 | 4.6 | 0.0319 | 1.74 (1.05,2.87) |
| Lipoprotein(a) (mg/L) |  |  |  |  |  |
| <42.95 | Ref |  |  |  |  |
| ≥42.95 | 0.34 | 0.25 | 1.82 | 0.1772 | 1.41 (0.86,2.32) |
| Homocysteine (μmol/L) |  |  |  |  |  |
| <15.7 | Ref |  |  |  |  |
| ≥15.7 | 0.7 | 0.29 | 5.75 | 0.0165 | 2 (1.14,3.54) |
| Alkaline phosphatase (U/L) |  |  |  |  |  |
| <104.5 | Ref |  |  |  |  |
| ≥104.5 | -0.33 | 0.29 | 1.33 | 0.2493 | 0.72 (0.41,1.26) |
| Albumin (g/L) |  |  |  |  |  |
| <43.25 | Ref |  |  |  |  |
| ≥43.25 | -0.15 | 0.25 | 0.36 | 0.5482 | 0.86 (0.53,1.4) |
| Total serum bilirubin (μmol/L) |  |  |  |  |  |
| <13.95 | Ref |  |  |  |  |
| ≥13.95 | 0.42 | 0.26 | 2.67 | 0.1022 | 1.52 (0.92,2.53) |
| Plasma fibrinogen (g/L) |  |  |  |  |  |
| <3.72 | Ref |  |  |  |  |
| ≥3.72 | -0.25 | 0.34 | 0.51 | 0.4754 | 0.78 (0.4,1.54) |
| D-dimer (ng/mL) |  |  |  |  |  |
| <146.22 | Ref |  |  |  |  |
| ≥146.22 | 1.2 | 0.26 | 21.09 | <0.0001 | 3.33 (1.99,5.57) |
| White blood cell count (×10⁹/L) |  |  |  |  |  |
| <10.55 | Ref |  |  |  |  |
| ≥10.55 | 1.34 | 0.38 | 12.67 | 0.0004 | 3.81 (1.82,7.94) |
| Neutrophil count (×10⁹/L) |  |  |  |  |  |
| <9.05 | Ref |  |  |  |  |
| ≥9.05 | 3.8 | 1.04 | 13.25 | 0.0003 | 44.65 (5.77,345.26) |
| Lymphocyte count (×10⁹/L) |  |  |  |  |  |
| <1.27 | Ref |  |  |  |  |
| ≥1.27 | -1.31 | 0.3 | 19.13 | <0.0001 | 0.27 (0.15,0.49) |
| Monocyte count (×10⁹/L) |  |  |  |  |  |
| <0.36 | Ref |  |  |  |  |
| ≥0.36 | -0.45 | 0.26 | 3.01 | 0.0827 | 0.64 (0.38,1.06) |
| Platelet count (×10⁹/L) |  |  |  |  |  |
| <207.5 | Ref |  |  |  |  |
| ≥207.5 | -1.12 | 0.27 | 17.29 | <0.0001 | 0.33 (0.19,0.55) |
| Mean platelet volume (f L) |  |  |  |  |  |
| <10.05 | Ref |  |  |  |  |
| ≥10.05 | 0.45 | 0.27 | 2.77 | 0.0963 | 1.57 (0.92,2.67) |

**Supplementary Figure S1-1:**

**Supplementary Material S1:**

**Supplementary Material S2:**

**Supplementary Material S3:**
